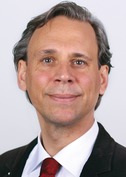# Towards Reliable and Quantitative Surface‐Enhanced Raman Scattering (SERS): From Key Parameters to Good Analytical Practice

**DOI:** 10.1002/anie.201908154

**Published:** 2020-02-20

**Authors:** Steven E. J. Bell, Gaëlle Charron, Emiliano Cortés, Janina Kneipp, Marc Lamy de la Chapelle, Judith Langer, Marek Procházka, Vi Tran, Sebastian Schlücker

**Affiliations:** ^1^ School of Chemistry and Chemical Engineering Queen's University Belfast BT9 5AG UK; ^2^ Laboratoire MSC Université Paris-Diderot 75013 Paris France; ^3^ Chair in Hybrid Nano-systems Nano-institute Munich Faculty of Physics Ludwig-Maximilians-Universität München 80539 Munich Germany; ^4^ Department of Chemistry Humboldt-Universität zu Berlin 12489 Berlin Germany; ^5^ IMMM—UMR 6283 CNRS Le Mans Université Avenue Olivier Messiaen 72085 Le Mans, Cedex 9 France; ^6^ CIC biomaGUNE and CIBER-BBN Paseo de Miramón 182 20014 Donostia-San Sebastian Spain; ^7^ Institute of Physics Faculty of Mathematics and Physics Charles University Ke Karlovu 5 121 16 Prague 2 Czech Republic; ^8^ Department of Chemistry and CENIDE University of Duisburg-Essen 45141 Essen Germany

**Keywords:** quantitative analysis, enhancement factor, Raman spectroscopy, SERS

## Abstract

Experimental results obtained in different laboratories world‐wide by researchers using surface‐enhanced Raman scattering (SERS) can differ significantly. We, an international team of scientists with long‐standing expertise in SERS, address this issue from our perspective by presenting considerations on reliable and quantitative SERS. The central idea of this joint effort is to highlight key parameters and pitfalls that are often encountered in the literature. To that end, we provide here a series of recommendations on: a) the characterization of solid and colloidal SERS substrates by correlative electron and optical microscopy and spectroscopy, b) on the determination of the SERS enhancement factor (EF), including suitable Raman reporter/probe molecules, and finally on c) good analytical practice. We hope that both newcomers and specialists will benefit from these recommendations to increase the inter‐laboratory comparability of experimental SERS results and further establish SERS as an analytical tool.

## Graphical abstract

**Parameters and pitfalls**: Key parameters and pitfalls that are often encountered in surface‐enhanced Raman scattering (SERS) are highlighted. Good analytical practice is proposed to increase the inter‐laboratory comparability of experimental SERS results and further establish SERS as an analytical tool.

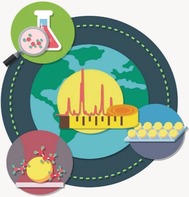

## 1 Introduction

**Figure 1:**
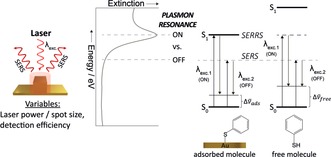
Key parameters in SERS experiments. Energy level diagrams for the plasmon resonance as well as the for molecular electronic resonances of the adsorbed and free molecule. In surface‐enhanced resonance Raman scattering (SERRS) λ_exc.1_ matches both the plasmon and the molecular (S_o_→S_1_) electronic resonance.

**Figure 2:**
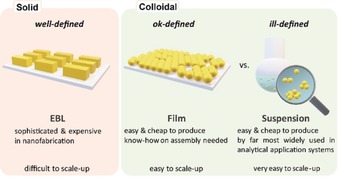
Major categories of SERS substrates for Raman signal enhancement.

Research in surface‐enhanced Raman scattering (SERS) is a rapidly growing field.^1^, ^2^ It ranges from fundamental theoretical studies to the development of new enhancing materials/substrates for real‐world applications. Thus, even for specialists, it is difficult to maintain a coherent view of the entire field. In part this may be due to differences in the priorities of researchers who come from different backgrounds. A large number of variables which occur in a typical SERS measurement (see Figure 1) makes it very difficult to compare the data from different studies. Herein, we would like to make suggestions that we hope will allow researchers to have their work compared with other approaches, thereby highlighting the analytical advancements in the field. We would hope that this could also be used to give non‐specialists an indication of which approaches might be most suitable for them and what performance they might expect to obtain. We begin by discussing ways in which the performance of SERS substrates can be reported, followed by highlighting the differences between solid and colloidal substrates (see Figure 2) as well as the challenge to thoroughly characterize nanoparticle suspensions. Finally, we give recommendations of what would be useful to support further development of SERS‐based chemical analysis in terms of good analytical practice.

## 2 Key Parameters in SERS and the SERS Enhancement Factor (EF)

As illustrated in Figure 1, at its simplest, the signal obtained in any SERS measurement is determined by four factors:

- The identity of the sample.
- A number of molecules present in the enhanced region which is being probed.
- The amplification provided by the substrate.
- Instrument settings and performance.

If we assume a known sample (i) with a defined Raman scattering cross‐section is being studied, this leaves just three factors which combine to give the observed signal. Although this apparently small number of factors seems relatively easy to compute and control, extracting a complete set of figures of merit in a SERS measurement is extremely challenging. This is partly because each of these factors is themselves determined by a combination of other variables and because some of the underlying variables are difficult to measure.

For example, in the variable (ii) the number of molecules in the enhanced region will depend on both the area of a surface within the probed volume and the surface coverage. Additionally, it is important whether the analyte has a high or low affinity for the enhancing surface since this determines the residence time of the analyte within the probed volume.

Similarly, in (iii) for the enhancing SERS substrate there are numerous theoretical and experimental studies^1^ that show the plasmonic enhancement provided by any substrate (typically Au, Ag, Cu or Al) is governed by a complex interplay of the material used, the morphology of the structure, and the coupling of excitation photons and the surface plasmon. It has also been demonstrated that semiconductor substrates (GaP, Si) can provide large near‐field enhancement and be used in surface‐enhanced spectroscopies.^3^

Finally, the instrumental factors in (iv) are a combination of the variables on the excitation side, such as the focusing conditions and the laser power, whereas on the detection side light‐throughput, spectral resolution (grating/spectrometer), and detection efficiency (CCD/CMOS) influence how the generated SERS signal is dispersed and how much of it is actually detected.

Overall, the above considerations highlight why it is so challenging to directly and objectively compare SERS results obtained in different laboratories worldwide. The most popular approach is to use an enhancement factor as a measure of the plasmonic enhancement and to define this in a way that aims to cancel out both the effects of instrumental factors and the intrinsic Raman scattering cross‐sections of the test molecule. This essentially provides a figure of merit of the effectiveness of the substrate and it can be transferred between laboratories.

This enhancement factor (EF), the so‐called “substrate enhancement factor”, is given by [Eq. (1)]^1^, ^4^

(1) $$EF=\frac{I_{SERS}}{N_{SERS}}\cdot \frac{N_{RS}}{I_{RS}}$$

with *I*_SERS_, the SERS signal, *I*_RS_, the classical Raman signal obtained without the SERS substrate, *N*_SERS_, the number of molecules excited in SERS and *N*_RS_, the number of molecules excited in classical Raman. The EF corresponds to the SERS intensity of one molecule divided by the Raman intensity of one molecule without the SERS substrate and can be seen as an absolute EF of the scattering cross‐section of the test molecule.

There are some fundamental issues with this approach which are discussed below but even before that it is useful to note that accurate estimation of the EF requires that each parameter in the Equation (1) is measured accurately. *I*_SERS_ is normally easy to measure as the SERS signal is normally large. The estimation of the three other factors is more critical.

First, since the normal Raman scattering (RS) cross‐sections of molecules are very low, it is necessary to make the *I*_RS_ measurement on a highly concentrated solution or on a crystal to be able to get a sufficient signal to noise (S/N) ratio. Thus, the measurement cannot be done on molecules having the same state as the ones excited in SERS. In addition, the Raman and the SERS spectra can also be different, as selection rules are modified due to specific interaction of the molecules with the substrate in SERS. It is important to use the same vibrational mode to compare signals in RS and SERS, as cross‐sections for different vibrations can differ greatly, and can experience different enhancement. This difference is mainly due to electronic interactions between the metal surface and the molecules.^5^ It is also referred to as the “first layer effect” as it appears only for the molecules in direct contact with the metal.^6^ In case of molecular electronic resonance (e.g., for dyes), it should be considered that the optical absorption cross‐section of molecules can change when interacting with metallic nanoparticles because of modification of the intrinsic polarizability of the molecule upon adsorption and molecule‐molecule interactions even at very low concentrations in the nm regime.^7^ In such a situation, the ignorance of (large) resonance shifts would lead to determination of an inaccurate SERS enhancement. To probe only the electromagnetic enhancement, it is possible to cover the metal surface with a thin dielectric layer (i.e. silica) as in the SHINERS approach.^8^ However, other problems could arise, notably when grafting molecules on the dielectric layer and because of the increased distance of the molecules from the nanoparticle surface.

Moreover, it is necessary to measure both *I*_SERS_ and *I*_RS_ under the same conditions, that is, the same excitation wavelength and intensity, the same focusing conditions, integration time and the same spectral resolution. Modern Raman instruments are nowadays often equipped with an EM‐CCD and users should be aware that the amplification (EM gain) might lead to issues in terms of quantification, especially at low signal levels.

Second, the estimation of the number of molecules that take part in SERS and in normal Raman is not trivial. For *I*_RS_, it requires the excitation and collection volumes in the solution or in the solid sample to be known. With the concentration or the density of the sample, the *N*_RS_ can then be estimated. *N*_SERS_ requires that the orientation and the surface coverage of the molecule be known. Assuming that the enhancement is localized on the metallic surface of the nanostructures, *N*_SERS_ can be obtained from a knowledge of the active surface area of the substrate that is being probed, the footprint of an adsorbed molecule (which can depend on orientation), and the surface coverage. Even if 100 % coverage is assumed, *N*_SERS_ can be difficult to determine accurately since the area can be difficult to define, or the interaction of the molecule with the surface may not take place as predicted. In the case of solid SERS substrates obtained by lithographic techniques, quantification of the surface area is facilitated by the periodic nature of the pattern, however, with colloidal nanostructures, the available area depends on the particle size and concentration. This means that the particle concentration used in a SERS experiment should be estimated and reported. Amorphous or disorganized rough noble metallic films obtained from metal sputtering or by adsorption of nanoparticles on surfaces are more challenging to characterize. Therefore, a consideration of the uncertainty of EF estimate in such a system is even more important.

A more difficult problem is that, while the SERS EF in principle gives the plasmonic enhancement independent of the probe molecule, in practice the measured number will typically depend on the surface affinity of the probe molecule. Usually, the surface coverage is not determined by independent methods, but concentrations of analyte are kept high and complete surface coverage is assumed. However, in reality, the adsorption of the analyte onto the surface depends very strongly on the interaction between the analyte and the surface. Often, to reach the surface, the analyte must replace a surfactant or penetrate a layer of a stabilizing species and consequently, the SERS response *I_SERS_* depends strongly on the affinity of an analyte to the nanoparticle surface. This means that for the same surface, strongly and weakly adsorbing analytes at the same concentration will give different surface coverages, with the result that the weakly adsorbing analyte will give smaller SERS signals. This can be misinterpreted as being due to different EF values, so that the EF will depend on the nature of the test molecule.

A solution could be the choice of a “neutral” analyte with potentially high binding affinity and the ability to displace the existing surface species or to co‐adsorb. In the literature, several probe molecules having high affinity to silver or gold have been proposed, for example, aromatic thiols including thiophenol or 4‐mercaptobenzoic acid, alkyl thiols (longer than C_5_ but not longer than C_16_), or molecules with an NH_*x*_ group, such as adenine. Such molecules have the advantages that they form an adsorbate on the metallic surface through S or N binding and they give characteristic SERS spectra that are easy to analyze. Dye molecules, such as crystal violet, methylene blue, or rhodamine 6G have also been used as they provide very high Raman cross‐sections because of resonant enhancement, albeit with lower affinity to the surface through electrostatic interaction.

Table 1 lists several potential SERS reporter/probe molecules that we consider to be useful, together with their respective advantages and disadvantages. We recommend the strongly adsorbing, non‐resonant molecules 4‐mercaptobenzoic acid (4‐MBA, deprotonated at pH 10) and adenine as standard Raman reporters. In the future, the community will need to agree which less‐strongly adsorbing compounds may also be suited to test accessibility of the surface of a SERS substrate. We emphasize that finding a single strongly binding standard is a necessary but not a definitive solution since it does not allow the accessibility of the surface to be measured. This is important because in most cases in which a particular analyte gives no signal with a reasonably plasmonically active substrate, it is because the analyte has not adsorbed onto the substrate rather than a problem with the plasmonic enhancement. Poor binding may be caused by many different reasons, most obviously it may just be that the structure of the analyte means that surface binding is not thermodynamically favorable. This can be overcome by modifying the surface^9^ but this is a topic not addressed herein. Alternatively, the reason for low signals may be that the substrate is plasmonically active, but the surface is passivated by a surface layer, for example protein which prevents access by all but the most strongly binding analytes. Such substrates will show large EFs with strongly binding test molecules but not be useful in practice since they will not allow detection of more weakly bound species. We, therefore, propose that in addition to very strongly binding test molecules, new substrates are also tested with weaker binding molecules so that the accessibility of the surface may be characterized. If this is done, then both the plasmonic efficiency and the ability to detect weaker binding molecules will be known and any compromises associated with a given substrate will be made apparent. For weakly adsorbing analytes, solid SERS substrates or ligand‐free nanoparticles (e.g., produced by laser ablation in liquids) might be preferable since they do not contain a capping agent on the surface. For strongly adsorbing analytes, SERS colloids with a capping agent are suitable as well.

**Table 1:** List of several potential SERS reporter/probe molecules. General requirements of the molecule: it adsorbs to both gold and silver surfaces; knowledge about the vibrational assignments, there are calculations and very solid work about all qualitative aspects of its SERS spectrum on different substrates.

Raman reporter	Advantages	Disadvantages
**SH‐functionalized molecules**	High affinity to gold surfaces, covalent binding	High water insolubility
		
*Recommended*: 4‐Mercaptobenzoic acid (deprotonated at pH 10) or the boronic acid equivalent	Polar aromatic thiol Good water solubility (pH 10) An attractive candidate as a universal probe and for modelling the surface	pH dependence of SERS fingerprint
		
Thiophenol	Small, well‐defined structure (conformation) High binding affinity relatively independent on the surfactant (citrate, CTAB) A good candidate as model analyte for most of the surfaces	Extreme toxicity and difficult handling (low vapor pressure, correct storage under inert gases)
		
1‐ and 2‐ Naphthalenethiol, 4‐Biphenylthiol	Strong, rich distinctive signal	Moderate toxicity
		
**N, NHx‐functionalized** **molecules**	Ideal test analytes for silver surfaces Good water solubility	pH dependence of surface coverage and of SERS fingerprint
		
*Recommended*: Adenine	A good candidate for non‐covalently binding models	Domination by one strong band mode (ca. 737 cm^−1^)
		
2,2′‐Bipyridine	Strong, rich distinctive signal	
		
Melamine	Strong, rich distinctive signal Relevant in the context of food adulteration	
		
**Dyes** ***(not recommended)***	Very strong SE(R)RS on silver and gold surfaces High water solubility	Change of optical cross‐section upon adsorption to metal
		
Rhodamine 6G, Crystal violet, Malachite green, Methylene blue	Mostly used analytes for testing new SERS substrates Raman reporter molecules in many applications	Strong electronic resonances High fluorescence background in RRS spectra (difficult to correctly determine EF, especially for Rhodamine 6G)
		
Riboflavin (vitamin B2)	Distinctive SERS spectrum Weaker electronic resonances Biological and food additive relevance	Weak surface adsorption Large size Low water solubility

Another way to estimate the SERS signal enhancement is to measure a relative EF. In this case, a reference sample is used to estimate a standard intensity (*I*_standard_) that is reproducible in any conditions and any environment. For simplicity and to be easy to obtain, such standard sample should be a solid sample as a Si surface with specific orientation and specific polarized excitation, a simple polymer such as polystyrene or an organic solvent.^4^ It can also be an internal standard inserted with the probe molecule as inherently included molecules on the SERS substrate^10^ or an isotopically labelled version of the molecular probe with a known relative concentration^4^, ^11^

The SERS intensity *I*_SERS_ could then be compared to this *I*_standard_ and the EF variation or optimization would be estimated by comparison with this *I*_standard_. This approach has the advantage of allowing the comparison of *I*_SERS_ measured with different instrumental conditions and equipment and also avoids the need to estimate the values of variables that are used to calculate the *N*_SERS_ and *N*_RS_. Such a methodology could be especially useful for inter‐laboratory and inter‐sample comparisons. For inter‐sample comparisons, the comparison should also be done using the same probe molecules (e.g. 4‐MBA, adenine), as the enhancement can include a contribution due to the chemical effect.

Considering all these parameters, drawing fair and quantitative comparisons between various SERS experiments is challenging. However, if all the details of the experimental conditions and the assumptions used in the calculation are given, especially those concerning the state of the molecules on the SERS substrate and on the reference sample used to measure the *I*_RS_ and *N*_RS_, there is no fundamental problem with using this approach to estimate the plasmonic efficiency.

## 3 Types of SERS Substrates and Their Characterization

SERS metallic substrates can be classified in three basic categories (Figure 2):^12^ 1) nanostructures fabricated directly on a solid substrate by lithography and template techniques in a top‐down approach^13^ 2) nanoparticles assembled and/or immobilized on solid substrates in a bottom‐up approach, and 3) nanoparticles in suspension (bare, Janus or core–shell of different compositions including silica‐, polymer‐coated, and shell‐isolated nanoparticles (SHINs)^8^ metal‐encapsulated dielectric core nanoparticles, bimetallic and core‐satellite configurations). Representatives from the three categories exhibit significant differences with respect to the homogeneity of their geometrical structure, instrumentation, and know‐how required for fabrication/synthesis, and the option to be scaled up. In addition to common metallic substrates, some semiconductors^14^ and 2D inorganic materials^15^ can provide reliable and quantitative SERS measurement.

The spectral reproducibility and the sensitivity of any new solid SERS substrate should be compared with commercially available substrates (Silmeco, Hamamatsu, ST Japan etc.) or standard home‐made substrates, such as nanoarrays prepared by nanosphere or colloidal lithography, metal film over nanospheres (MFON), or thin metal film (4 nm) on glass.^16^ Also new colloidal SERS substrates should be compared with commercially available substrates (BBI, nanoComposix, Nanopartz, NanoWerke etc.) or standard home‐made 20–50 nm gold/silver colloids prepared by the Turkevich (Au, citrate) or Leopold/Lendl (Ag, hydroxylamine) method.^17^ Although silver is generally plasmonically more active than gold, oxidation is a problem. In the case of home‐made solid and colloidal SERS substrates, detailed preparation protocols should be provided.

The SERS substrate plays a crucial role as the electromagnetic enhancement is related to the material, size, and shape of the nanostructure.^1^, ^18^ Currently, considerable effort continues to be devoted to the preparation of novel and ever more sophisticated SERS substrates. While theoretical analysis and modelling of their electromagnetic properties can underpin their rational design and fabrication it is also critical that standardized methods are used in their characterization. In particular, electron microscopy and extinction/reflectance spectroscopy can provide an idea of the shape, size distribution, and plasmon resonance for metallic nanostructures. For substrates on a solid support, scanning electron microscopy (SEM) and scanning probe microscopies, such as atomic force microscopy (AFM), scanning tunneling microscopy (STM), and scanning near‐field optical microcopy (SNOM) can provide valuable information on the structures created. However, while such measurements are often the only ones that are reported for novel substrates, this is typically not sufficient to predict their SERS performance. The central problem is the dramatic spatial variations in local optical fields within a few nanometers that are predicted by theory and observed in experiments which make the enhancement hugely dependent on very small structural features. These can be studied by other methods, as discussed below but it means that simple imaging of a substrate is not a good guide to the SERS enhancement that it may produce and it should not be used for this purpose. Conversely, local optical fields can be probed by excitation of the plasmon with an electron beam in the highly regular arrangements on nanostructures fabricated on solid supports and at the single‐aggregate level with great precision by excitation of the plasmon with an electron beam. Electron energy loss spectroscopy (EELS) and optically excited local field intensities can differ, so both EELS and optical excitation could be used for plasmonic characterization. By EELS, shifts of plasmon resonances with small changes in gap size can be mapped.^19^ We recommend combining several characterization techniques in a correlative approach on the same sample. This also facilitates the comparison with predictions from computer simulations.

**Figure 3:**
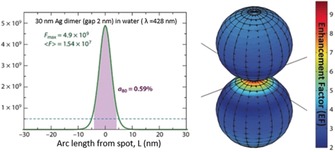
Calculated enhancement factor (EF) distribution around the hot spot in a dimer of silver spheres (radius 30 nm, gap 2 nm). The excitation wavelength is taken at the dipolar localized surface plasmon resonance, which provides the maximum SERS EF. The logarithmic false color maps show graphically the surface SERS EF distribution in the |E|^4^ approximation. The plot on the left shows the SERS EF in the plane of incidence as a function of arc length *L* along the surface together with the maximum SERS EF (*F*_max_), the surface‐averaged SERS EF (⟨F⟩) and the relative area a_80_ from which 80 % of the total SERS signal originates. Figure adapted from Ref. 21.

For colloidal SERS substrates, transmission electron microscopy (TEM) can be used to obtain information on nanoparticle size distribution and shapes, provided that a proper statistical evaluation is performed. Dynamic light scattering (DLS) can be particularly useful for nanoparticles of narrow size distribution, and for an assessment of nanoaggregate formation when analyte molecules are added. The potential interaction with analyte molecules can be studied by determining the zeta potential. Although usually provided by the SERS spectrum itself, X‐ray photoelectron spectroscopy (XPS) and electrochemical approaches can be used to evaluate the interactions at the interface between the plasmonic nanostructure and the molecules to greater detail. Importantly, the interaction with light can be simulated for most of the cases, giving insight into, for example, the spectral response, electric‐field confinement, and polarization‐dependence. However, the information provided by these characterization techniques depends on the system under study and should be judged critically. The first question that we need to keep in mind—and try to answer using these methods and techniques—is: where is the signal in our SERS measurements coming from? It is generally accepted that the SERS signal predominantly arises from molecules located in very small (few nm^3^ or even less^20^) regions with extremely high local electric field, so‐called hot spots. A simple calculation of an elongated Ag nanoparticle shows that 12 % of the total surface area is responsible for 80 % of the collected SERS signal. These numbers can be even more striking when considering Ag sphere dimers with a gap of 2 nm (see Figure 3, right). In that case, only 0.59 % of the total surface contributes to 80 % of the measured SERS signal (see Figure 3, left).^21^ For this reason, it is important to keep in mind that what we are seeing, sensing, targeting, studying in SERS is usually a tiny fraction of the total adsorbed molecules.^22^ The highly localized nature of the hot spots forces us to rethink the validity of the standard characterization methods for colloids or nanostructured substrates when trying to link them to their SERS performance. This strong dependence on nanoscale inhomogeneities makes it challenging to predict enhancement factors or reproduce results obtained by different groups using similar colloids or substrates. Our characterization efforts should be centered on accessing to these regions/molecules to fairly reproduce results and correlate them with theory/simulations.

Overall, we strongly recommend that the plasmonic characterization must be carried out at the level of individual nanoparticles and nanoaggregates by correlative techniques based on a combination of electron microscopy, dark‐field and SERS microspectroscopy on the same sample.^23^ Generally, the optical characterization should be performed before electron microscopy owing to potential sample damage caused by electron microscopy. Commercial providers offer, for example, instrumentation for correlative Raman/SEM (for example, Raman: WITec, SEM: Tescan and Zeiss).

## 4 Good Analytical Practice

In this Section, we point out to some of the analytical shortfalls that in our opinion should not be further perpetuated. In SERS‐based quantification of analytes, a calibration model of the dependence of a SERS signal on the concentration of an analyte is typically used to predict the concentration of this analyte in an unknown sample. We strongly recommend that in addition to the calibration procedure also a validation step is performed to make sure the SERS detection can do more than making accurate predictions on the set of data that it was calibrated with.^24^ In other words: a SERS‐based detection scheme without a validation step should be questioned regarding its relevance to an analytical application.

Most SERS studies devoted to demonstrating analyte quantification capability tend to focus on the ultra‐sensitivity of SERS through the lens of the limit of detection (LOD) and sometimes limit of quantification, namely the lowest concentrations that the sensor is capable of detecting and quantifying, respectively. However, more important than the LOD is the ability to predict concentrations with accuracy in the range of concentrations relevant to the actual concentration likely to be encountered in the targeted samples. Therefore, sensor development should aim for the concentration range that matches the specific quantification problem at play, for example, a specific physiological concentration in bio‐detection.

It is useful to note that the LOD is often used as a means of characterizing the performance of novel SERS substrates. While this is valid if the objective is to demonstrate detection of a specific target analyte, it is more often used as a simple general indication of SERS performance. The obvious problem with this approach is that the LOD depends not only on the substrate used and the sample concentration but also on instrumental factors, such as the collection efficiency or the grating efficiency and detector sensitivity. Thus, high‐performance instruments will give higher signal to noise ratios than lower ones with the same sample. This means that LOD comparisons should really be limited to side‐by‐side comparisons of substrates. For inter‐laboratory comparisons the instrument performance should also be reported, for example by showing the normal Raman spectrum of a standard solvent along with clearly stated experimental conditions.

In addition to the sensitivity range, two other indicators of performance that are routine in the evaluation of chemical analysis methods should be reported. First, the recovery rate, which is the ratio of the detected concentration to the actual concentration in the sample; this allows estimating whether the sensor is overestimating or underestimating the actual analyte concentration. Second, the root mean square error (RMSE) of prediction, which gives an estimate of the precision of the SERS sensor's readout, should be determined.^25^ For maximal relevance to the application, this last figure of merit is best calculated on the validation data set to test the predictive power of the sensor on samples which are known to the experimenter but unknown to the calibration process.

A significant issue with quantitative SERS measurements is the reproducibility. For colloids this is typically equated with the extent to which different preparations of nominally the same particles give the same absolute SERS intensity; for solids this should be linked with signals given by different production batches, but it is often confused with uniformity, that is, the signal given at different points on the same surface. It is useful to have an indication of variation both within and between batches and we would recommend in case of colloidal nanoparticles in suspension to average the results of three different batches and at least 10 individual measurements per batch under the same conditions (when long integration times of ca. 10 s are needed) and ideally more than 100 individual measurements (for short integration times around 1 s). In the case of assemblies or other solid substrates, depending on the complexity of the fabrication and uniformity of the materials, measurements of tens of points on a surface can give an approximate indication of the uniformity of a substrate; to obtain statistically significant results SERS data should be averaged from 3–5 samples with a few 100 s of individual measurements per sample recorded under the same conditions (typically short integration times <1 s) and distributed over the reported area.^26^

It is important to note that there are significant limits to the application of SERS to complex real‐world samples in which there may be issues with interference from the non‐target constituents. Often, switching to “real‐world” samples from “clean‐laboratory” samples introduces the problem of interference from impurities in the sample matrix. As examples, blocking of the SERS substrate surface or changed plasmonic properties of the substrate, or strong SERS signals from other compounds should be mentioned. This illustrates the importance of realistic sample matrices also in the laboratory setting. Such problems may be reduced by pre‐treating the sample to remove interfering species^27^ or by chemically modifying the surface of the SERS substrate, for example, with self‐assembled monolayers, or even with species that promote binding of the target species.^9^, ^28^ Such approaches improve selectivity at the cost of the general applicability of a SERS sensor.

As discussed above, changes in the EF of the substrate in the presence of analyte species must be properly addressed, specifically in quantification. Of course, having reasonably reproducible substrates is helpful, as is using a sampling method designed to minimize variation. For example, the incubation of SERS substrate in a solution containing analyte yields SERS signal with a lower variability than the droplet deposition.^29^ However, these precautions may still not be sufficient to prevent variations owing to the sample matrix effects.

Fortunately, EF variations can be mitigated by the addition of a standard whose response will change in the same way as the target to experimental variations. The target signal can then be normalized to the internal standard signal, see the Review article Ref. 30. The most obvious internal standards are isotopologues of the target since these will track the variation in the EF of the target very well.^31^ Alternatively, chemically similar compounds may be appropriate^32^ or standard addition can be used.^33^ A further approach is the rational design and synthesis of SERS nanotags (typically core–shell nanoparticles) where the standard (SERS‐active label) can be embedded directly between core and shell such as in core‐molecule‐shell (CMS) nanoparticles.^34^ An additional advantage of the introduction of internal standards is that it dramatically reduces the need for rigorous batch‐to‐batch reproducibility of substrates and spectral acquisition. We, therefore, would like to emphasize that by using an appropriate internal intensity standard quantitative SERS analysis can be achieved even without very highly reproducible substrates.

Finally, we would like to point out that the presented attempt on formulating recommendations for the practice of SERS is certainly just a starting point for future discussions in the community and that we are looking forward to feedback from our colleagues.

## Conflict of interest

The authors declare no conflict of interest.

## Biographical Information

*Steven E. J. Bell received his PhD from Queen's University Belfast (with Prof. John McGarvey) and worked at the Rutherford‐Appleton Laboratory and the University of York before returning to QUB where he is a Professor of Physical Chemistry. His research centers on nanomaterials and Raman spectroscopy. He has a particular interest in the application of Raman methods to real world problems including medical and security applications and was founder/director of a successful company manufacturing Raman spectrometers*.

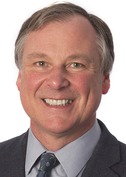

## Biographical Information

*Janina Kneipp received her diploma and doctorate (with Prof.  Dieter Naumann) at Freie Universität Berlin and Robert‐Koch‐Institut. She worked at Erasmus Universiteit Rotterdam*, *at Princeton University's Chemistry Department, and at BAM in Berlin before being appointed at Humboldt‐Universität zu Berlin in 2008. The focus of her research lies in the development of sensitive vibrational methods for studies of complex microstructured materials, multiphoton‐excitation, and plasmon‐supported nanospectroscopy*.

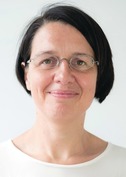

## Biographical Information

*Marc Lamy de la Chapelle got his PhD in science physics in 1998 at the University of Nantes (with Prof. Serge Lefrant). He is now professor at the Université du Mans (Institute of Molecules and Materials of Le Mans UMR6283) and was director of the CNRS French network on molecular plasmonics and enhanced spectroscopies (2011–2018). His research activities are focused on nano‐optics, plasmonics, and SERS. He develops SERS sensors to detect pollutants or to study biological media*.

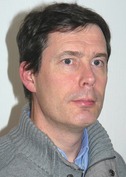

## Biographical Information

*Sebastian Schlücker received his Dr. rer. nat. (PhD) in physical chemistry from Würzburg University (with Prof. Wolfgang Kiefer) in 2002. After postdoctoral studies at NIH in Bethesda/MD and his Habilitation, he became Associate Professor of experimental physics at Osnabrück University in 2008. Since 2012 he is Full Professor of physical chemistry at the University Duisburg‐Essen. His major research interests are the physics and chemistry of molecularly functionalized plasmonic nanostructures and their application in biomedicine as well as in chemical energy conversion*.